# Longer Duration of Cord Clamping Improves Nicu Survival Without Major Morbidities in Very Preterm Infants

**DOI:** 10.3390/children11121546

**Published:** 2024-12-20

**Authors:** Priya Jegatheesan, Esther Belogolovsky, Matthew Nudelman, Sudha Rani Narasimhan, Angela Huang, Balaji Govindaswami, Dongli Song

**Affiliations:** 1 Division of Neonatology, Department of Pediatrics, Santa Clara Valley Medical Center, San Jose, CA 95128, USA; sudharani.narasimhan@hhs.sccgov.org (S.R.N.); angela.huang@hhs.sccgov.org (A.H.); balaji.swami@me.com (B.G.); dongli.song@hhs.sccgov.org (D.S.); 2 Department of Pediatrics, School of Medicine, Stanford University, Stanford, CA 94305, USA; estherb@stanford.edu; 3 School of Medicine and Public Health, Department of Medicine and Clinical Informatics, University of Wisconsin Hospital and Clinics, Madison, WI 53210, USA; matthew.nudelman@hhs.sccgov.org; 4 Valley Health Foundation, San Jose, CA 95128, USA

**Keywords:** delayed cord clamping, deferred cord clamping, duration of cord clamping, umbilical cord management, very preterm infants, preterm infant outcomes, survival without major comorbidities

## Abstract

Background: Longer duration of deferred cord clamping (DCC), at least 120 s, is associated with the highest reduction in mortality compared to shorter durations of DCC or immediate cord clamping in preterm infants. We compared the neonatal outcomes of very preterm infants who received at least 60 s to those who received at least 120 s of DCC. Methods: This is a retrospective single-center study including preterm infants born <33 weeks of gestational age (GA) between 2014 and 2019. The intended duration of DCC was 60 s in Period 1 (January 2014 to June 2016, *n* = 139) and 120 to 180 s in Period 2 (July 2016 to December 2019, *n* = 155). We compared the demographics, delivery room measures, and neonatal outcomes between the two periods as intent-to-treat analysis and per protocol analysis. Results: The intended duration of DCC was completed in 75% of infants in Period 1 (*n* = 106) and 76% of infants in Period 2 (*n* = 114). There was an increase in survival without major morbidities in the infants that received at least 120 s of DCC, which remained significant after adjusting for GA and erythropoietin use (Odds ratio 8.6, 95% CI 1.6 to 45.7). Conclusions: Longer duration of DCC is associated with improved survival without major morbidities in preterm infants <33 weeks GA.

## 1. Introduction

Deferred or delayed cord clamping (DCC) increases blood transfusion from the placenta to the newborn [1,2,3] and improves hemodynamic stability and cardiovascular transition at birth. In preterm infants, DCC for at least 30–60 s has been shown to reduce mortality, risks of transfusion, inotropes for low blood pressure (BP), intraventricular hemorrhage (IVH), and improved hematocrit and BP after birth [4,5,6,7]. National organizations, including the American College of Obstetricians and Gynecologists, American Academy of Pediatrics, and Neonatal Resuscitation Program (NRP), have recommended at least 30–60 s of DCC for preterm infants. International organizations, including the World Health Organization [8], European Resuscitation Council [9], Neonatal Resuscitation group of the Spanish Society of Neonatology [10], and Canadian Fetus and Newborn Committee [11], have recommended at least 60 s of DCC for preterm infants.

Early studies have shown that increasing the time of cord clamping from birth leads to incremental increases in the blood volume transfer from the placenta to the newborn. In term infants, the percentage of feto-placental blood volume in the infant is 67% at birth, which increases to 80% and 87% when the cord is clamped at 1 and 3 min after birth [1]. Saigal et al. have shown that with 5 min of DCC, there is up to 50% increase in blood volume from birth in both term and preterm infants [3]. However, the rate of placental transfusion with DCC is slower in preterm infants than in term infants; within the 1st minute of DCC, 75% of the placental transfusion occurs in term infants, whereas only 50% in preterm infants. This suggests that preterm infants may need a longer duration of DCC to maximize the benefits of placental transfusion. Randomized controlled trials (RCT) have shown that DCC 30–180 s improved preterm neonatal outcomes compared to immediate cord clamping. An individual patient data (IPD) network meta-analysis including 47 trials with 6094 preterm infants compared different durations of DCC and showed that long DCC duration (≥120 s) has the highest reduction in mortality by 69% [7]. None of the individual studies included in this network meta-analysis directly compared 60 s to 120 s DCC.

DCC is beneficial to preterm infant outcomes, and a longer duration of DCC is more beneficial in reducing mortality. However, the ideal duration of DCC has still not been agreed upon. Physiologically, the cord should be clamped after respiratory function is established. This process may take longer or may not be achievable without respiratory support in some preterm infants. Hence, there are concerns that prolonging the DCC duration may delay resuscitative efforts in infants who are not breathing. Our center implemented 30 s of DCC as the standard of care for preterm infants in 2007 and increased DCC duration to at least 60 s in 2011. We evaluated the neonatal outcomes in preterm infants who received 30–45 s vs. 60–75 s DCC and demonstrated that longer duration was associated with a reduction in hypothermia, transfusions, and intubation [12]. In 2016, we increased the duration of DCC to at least 120 s in infants who are breathing by 60 s to further increase placental transfusion. This study’s objective was to compare the neonatal outcomes of preterm infants born <33 weeks of gestational age (GA) who received 60 s DCC to those who received ≥120 s DCC.

## 2. Methods

### 2.1. Study Design and Subjects

This is a single-center retrospective, observational study and data collection was approved by the institutional review board (IRB, IRB #11-033ER) as a quality improvement project.

**Figure 1:**
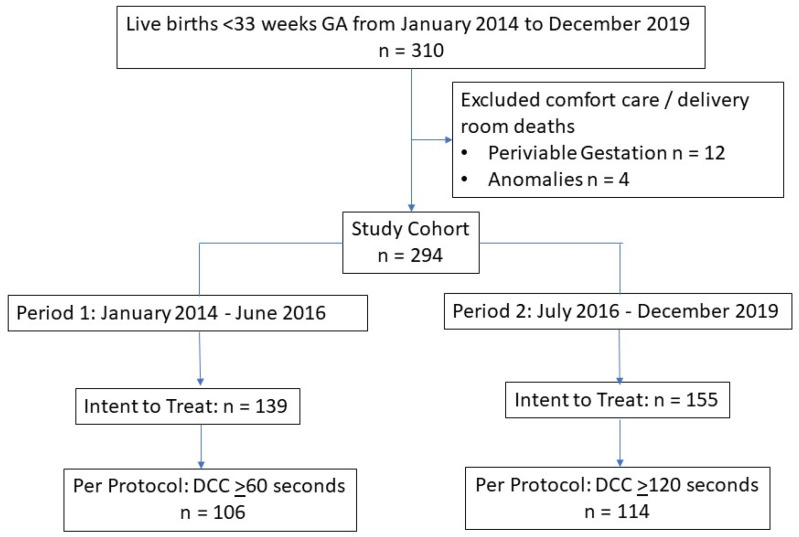
Enrollment.

This study was performed at an AAP Level IV NICU in a California public safety-net hospital from January 2014 to December 2019. The intended duration of DCC at birth was ≥60 s from January 2014 to June 2016 (Period 1) and ≥120 s from July 2016 to December 2019 (Period 2). This study included preterm infants born at <33 weeks GA (Figure 1). Infants with no intent to resuscitate or those who died in the delivery room were excluded.

### 2.2. Standardized Delivery Room Management Bundle and NICU Care for Very Preterm Infants

Our delivery room (DR) management bundle for very preterm infants includes pre-delivery huddles and role assignments with one provider designated to care for the baby during DCC in both vaginal and cesarean deliveries. The details of this bundle have previously been published [13]. Our center has performed at least 60 s DCC as part of this standardized DR management bundle for all preterm infants since March 2011 [12]. The intended DCC duration was incrementally increased to at least 120 s in July 2016 and 180 s in January 2018 for those infants who were breathing by 60 s of life.

During DCC, the infant was placed on a portable warming mattress and gently stimulated by tactile stimulation. Oral and nasal airways were suctioned using a bulb syringe as needed. If the infant was breathing spontaneously or in response to stimulation and suctioning by 60 s, then DCC was continued to the intended duration. DCC was discontinued at any time if there were concerns for placental abruption with active bleeding per the assessment of the obstetrician at the delivery, placenta detaching, cord avulsion/tear or tight knot disrupting of placenta-infant circulation, if the infant appeared lifeless without tone or heart rate, or if the infant was persistently apneic despite stimulation and bulb suctioning. In 2016, cord milking was standardized when the intended duration of DCC was not performed. Cord milking was performed by the obstetric team when feasible. In June 2019, cord milking was excluded in extremely preterm infants <29 weeks GA based on the early evidence from a RCT that showed cord milking increased the risk of severe intraventricular hemorrhage (IVH) in these extremely preterm infants [14].

After DCC was completed, all preterm infants started with continuous positive airway pressure (CPAP) in the DR as per standard of care. The FiO_2_ at the initiation of CPAP of 5 cmH_2_O was 30% for infants <29 weeks GA and 21% for infants born between 29 and 32 weeks GA. APGAR score was assigned by the NICU provider while the infant was still connected to the cord at 1 min of life. We have a comprehensive standardized care bundle for very preterm infants in the NICU that includes enteral and parenteral nutrition guidelines such as exclusive human milk feeding until after 34 weeks postmenstrual age (PMA), early colostrum oral care, indications for insertion and removal of central line, intubation criteria and respiratory care with CPAP, non-invasive or invasive mechanical ventilation, caffeine, Vitamin A prophylaxis for prevention of chronic lung disease (CLD), IVH prevention bundle, high humidity protocol to maintain euthermia for the extremely preterm infants, criteria for blood transfusion, use of erythropoietin (EPO) for extremely preterm infants, phototherapy guidelines for hyperbilirubinemia, and conservative approach for evaluation and treatment of patent ductus arteriosus. The anemia prevention bundle was expanded to include routine use of EPO for infants >28 weeks PMA who were anemic in June 2016, along with at least 120 s of DCC to reduce packed red blood cell (RBC) transfusion risk. In April 2017, less invasive surfactant administration was implemented for those infants who were not intubated and needed surfactant.

### 2.3. Data Collection

Demographics, DR measures interventions and outcomes during NICU stay were obtained from the NICU database. Hematocrit values were obtained retrospectively from medical records. Duration of DCC was documented in seconds throughout the study period. Cord milking and breathing before cord clamping were standardized as part of our documentation in 2016 and were obtained only for Period 2.

Demographic variables included GA, birthweight (BW), cesarean delivery, antenatal steroids (ANS), and maternal magnesium; DR measures included completion of the intended duration of DCC (at least 60 s during Period 1 and at least 120 s during Period 2), DCC duration in seconds, chest compressions, and intubation; NICU interventions included early RBC transfusion (within 72 h of life), any RBC transfusion during NICU stay, hematocrit values at 0–2 and 12–24 h of life, and EPO use; and neonatal outcomes included severe IVH (grade 3 or 4), late onset sepsis (LOS, positive blood or cerebral spinal fluid culture at >72 h of life), CLD (requiring oxygen/respiratory support at 36 weeks PMA), severe retinopathy of prematurity (ROP ≥ stage 3, or plus disease, or received anti-VEGF/laser treatment), necrotizing enterocolitis (NEC, Bell stage ≥ 2, survival without major morbidity (severe IVH, NEC, LOS, CLD, severe ROP), and death.

### 2.4. Analysis

Demographics, DR and NICU measures and interventions, and neonatal outcomes were compared between infants born during Periods 1 and 2 with intent to treat (ITT) analysis. Per protocol (PP), an analysis was performed comparing infants who received at least 60 s of DCC from January 2014 to June 2016 and those who received at least 120 s of DCC from July 2016 to December 2019. Both ITT and PP analysis were performed to compare the 2 groups using generalized estimating equations. STATA 14.0 (Statacorp, College Station, TX, USA) was used for statistical analysis. A *p*-value < 0.05 was considered significant. Risk-adjusted analysis, adjusting for GA, was performed for outcomes that were different between the two periods.

## 3. Results

This study included 294 preterm infants born <33 weeks of gestation (Period 1: *n* = 139, Period 2: *n* = 155). There were no differences in baseline demographics between the two periods (Table 1). The intended duration of DCC was successfully achieved in 220 infants (Period 1: *n* = 106, Period 2: *n* = 114) (Figure 1).

### 3.1. Deferred Cord Clamping Implementation

Overall, 75% of infants received the intended duration of DCC; there was no difference in the percentages of infants who received the intended duration of DCC between Periods 1 and 2 (Table 2). The distribution of DCC duration is shown in Table 2. The median duration was 60 s in Period 1 compared to 129 s in Period 2. While there was no difference in the percentage of infants who completed the intended duration of DCC, there were more infants who had DCC discontinued at <60 s in Period 1 compared to Period 2 (24% vs. 14%, *p* = 0.04). The reasons for discontinuing DCC are shown in Table 2. There were no differences in the reasons for discontinuing DCC between the two periods. In Period 2, 93% of infants were breathing before cord clamping.

### 3.2. Delivery Room and NICU Measures and Interventions

The DR and NICU measures and interventions are shown in Table 3A. There were fewer infants with 1 min APGAR < 4 in Period 2 compared to Period 1, although there were no differences in the 5-min APGAR scores. In the ITT and PP analysis, there were no differences in hypothermia, chest compressions, DR intubation or any intubation, hematocrit values, or early or any RBC transfusions between Period 1 and Period 2. There was an increase in the use of EPO in Period 2 compared to Period 1 in both the ITT (45% vs. 11%; *p* < 0.001) and PP (43% vs. 9%; *p* < 0.001) analysis.

### 3.3. NICU Mortality and Morbidities

Neonatal mortality and morbidities are shown in Table 3B. In both ITT and PP analysis, there were no differences in severe IVH, LOS, CLD, severe ROP, NEC, or death between Period 1 and Period 2. Survival without major morbidities increased from 76% in Period 1 to 88% in Period 2 (*p* = 0.032) in PP analysis. This increase remained significant after adjusting for GA and EPO use (Odds ratio 8.6, 95% CI 1.6 to 45.7) (Table 4). In subgroup analysis based on GA, this increase in survival without major morbidities was seen in the ≥29 weeks GA (87% vs. 97%, *p* = 0.038) but not in the <29 weeks GA (44% vs. 59%, *p* = 0.3).

## 4. Discussion

Our single-center observational cohort study showed the feasibility of extending the duration of DCC to at least 120 s in 75% of the very preterm infants born at <33 weeks GA. Extending the DCC duration to at least 120 s is associated with increased survival without major morbidities in these infants.

### 4.1. Benefits with Longer Duration of Cord Clamping

The benefits of deferred cord clamping in very preterm infants have been shown in RCTs. However, the optimal duration of DCC has not yet been agreed upon. Cumulative evidence indicates that a longer duration of DCC may provide more benefits. Early placental transfusion studies showed an incremental increase in newborn blood volume with delaying cord clamping up to 5 min [2,3]. DCC duration was extended to 2–3 min in multiple RCTs, showing an increase in hematocrit levels with no associated adverse effects in moderately preterm infants between 30 and 36 weeks GA [15,16,17,18]. A 2023 IPD network meta-analysis compared different durations of DCC and showed that the long DCC ≥120 s reduced mortality by 69% [7]. Of the 47 studies included in this meta-analysis, only 5 RCTs included DCC ≥120 s; 2 of these studies [16,17] excluded infants who needed resuscitation, and 3 studies [19,20,21] had a trolley to provide positive pressure ventilation (PPV) if an infant needed resuscitation during DCC. A more recently published RCT, the VentFirst trial [22], evaluated the effect of ventilation during DCC on the outcomes of extremely preterm infants. The infants were randomized to 60 s DCC with tactile stimulation or 120 s DCC with CPAP or PPV as needed. This study demonstrated that in the subgroup of infants who were not breathing well, the 120 s DCC with ventilatory support group had better APGAR scores at 5 min, lower DR intubation, and higher hematocrit values in the first 24 h of life.

In our center, we increased the duration of DCC in a stepwise fashion over the last decade to optimize the outcome in preterm infants. We initiated 30–45 s of DCC in late 2007 and then increased it to 60–75 s when neonatal resuscitation program recommendations included 60 s of DCC in 2011. Previously, we have shown that at least 60 s of DCC compared to at least 30 s of DCC reduced the risk of admission hypothermia, intubations, and RBC transfusions [12]. In 2016, to optimize the transition of preterm infants and placental transfusion, we deferred clamping of the cord to 120 s if the infant was breathing by 60 s. If the infant was not breathing by 60 s despite warmth, drying, stimulating and suctioning, then we clamped the cord as we did not have a system to provide PPV during DCC.

### 4.2. Feasibility

The duration of DCC in preterm RCTs has varied from 30 to 180 s. In our study, it was feasible to implement 60 to 120 s of DCC in 75% of the infants successfully without an increase in adverse effects in the delivery room, including hypothermia, chest compressions, and DR intubation. This is similar to the large Australian Placental Transfusion study [23], including <30 weeks GA preterm infants in which 74% of their cohort randomized to the DCC group received at least 60 s DCC. Two studies including more mature infants have shown that more than 85% of their infants received ≥120 s DCC after excluding infants who required resuscitation, 86% in <34 weeks GA by Rana et al. and 88% in 30–36 weeks GA by Ranjit et al. [16,17]. A recent IPD network meta-analysis of 47 trials with 6094 preterm infants comparing the different durations of cord clamping (15–45 s short DCC, 45–120 s medium DCC, and ≥120 s long DCC) showed that the adherence to short and medium DCC intervention was 80% and long DCC was 67%. There were two RCTs that included preterm infants <32 weeks GA receiving ≥120 s DCC and <30 weeks GA receiving ≥180 s DCC while providing immediate positive pressure support with intact cord in their intervention group and showing long DCC was successfully performed in 60% and 75% of the infants respectively [19,21]. It is interesting that only 60–75% of infants who received ≥120 s of DCC even with respiratory support before cord clamping. In our study, we show that in a non-clinical trial setting, with standardized guidelines on continuing DCC beyond 60 s in an infant with respirations, we were able to successfully perform at least 120 s of DCC in 75% of all preterm births <33 weeks GA. The Vent First study [22] has shown that only the infants who were not breathing well benefited from providing ventilatory support during 120 s DCC. Having a system to provide ventilatory support with intact cord for very preterm infants not breathing with tactile stimulation in the first 60 s will equip centers with the ability to provide ≥120 s DCC in >85% of infants. Once there is an opportunity to provide respiratory support with intact cords for those infants who are not breathing well, maternal bleeding concerns will be the limitation for a longer duration of DCC.

### 4.3. Safety

In our study, there was no difference in the DR measures of 5-min APGAR, hypothermia, DR intubation, or chest compressions between the 2 periods. We developed a standard protocol with clearly defined indications for when to discontinue DCC. When there is a concern for maternal bleeding, the obstetricians can decide to clamp the cord at any time. If the infant is not breathing by 60 s with warmth, drying, tactile stimulation, and suctioning, the pediatric providers can decide to stop DCC at any time. Our experience shows that prolonging the duration of DCC to at least 120 s did not have an adverse effect on the DR measures in our study.

### 4.4. Transfusion Benefit

Improved placental transfusion is one of the potential benefits of a longer duration of DCC. We have previously shown that 60–75 s of DCC is associated with an increase in hematocrit values and decreases in early (within 72 h of life) or any RBC transfusion during NICU stay when compared to 30–45 s of DCC [12]. In this study, we compared the 60-s DCC cohort to the 120-s DCC cohort and showed that there is no difference in hematocrit values, although there is a suggestion of reduction in RBC transfusions. Previous RCTs and meta-analyses have shown that DCC 30 to 180 s, compared to early cord clamping (ECC), is associated with higher hematocrit values and a lower risk of RBC transfusion [4,5,6,15,16,17,18,24,25]. However, the RCTs have not directly compared RBC transfusion between 60 and 120 s DCC. The recent IPD network meta-analysis comparing short, medium, and long DCC showed that short and medium DCC compared to ECC decreased the risk of transfusions, although the evidence for long DCC was inconclusive due to insufficient events for comparison.

### 4.5. Neonatal Outcomes

RCTs and meta-analysis of RCTs in preterm infants have shown that there is a significant reduction in mortality in those who receive DCC compared to ECC [4,5,6,7,23] and reduction in IVH [4]. The duration of DCC in these RCTs ranged from 30 to 120 s, and comparisons were made between the DCC and ECC groups. In the Australian placental transfusion study, there was no difference between the ECC group and 60 s of the DCC group in the composite outcome of survival without major morbidities [23]. A 2018 meta-analysis, including 27 trials and 2384 preterm infants, performed subgroup analysis based on the duration of DCC (≥30–45 s, ≥45–60 s, ≥60–120 s, and ≥120 s) and did not see any differences in any of the neonatal outcomes, IVH, CLD, NEC, LOS, or ROP [5]. However, a more recent 2023 IPD network meta-analysis, including 47 trials and 6094 infants, compared short (14–45 s), medium (45–120 s), and long (≥120 s) DCC showed that long DCC ranked highest in reducing mortality, but showed no difference in IVH. We performed both ITT and PP analyses to compare the outcomes between the two study periods and observed an increase in survival without major morbidities that were significant in the PP analysis. While increasing DCC duration from 60 to 120 s did not make a difference in individual neonatal outcomes (IVH, LOS, CLD, ROP, NEC, Death), small non-significant trends across multiple morbidities likely contributed cumulatively to the observed improvement in the composite outcome of survival without major morbidities. In our study, this increase in survival without major morbidity is mainly contributed by the preterm infants born between 29 and 32 weeks GA, although there is a similar trend noted in the <29 weeks GA subgroup as well. This difference between the results of the GA subgroups is likely related to the smaller sample size in the younger GA infants in our study cohort. We had an increase in the use of EPO in the 120 s DCC cohort as part of our bundle for eliminating transfusions. However, this improvement remained after adjusting for the use of EPO. This is consistent with the finding of the RCT that using EPO in extremely preterm infants did not make a difference in death and neonatal outcomes except for an improvement in the transfusion rate [26]. Our study is an observational cohort study and could be at risk for selection bias, although the rate of DCC was similar between the two study periods at 74–76%.

DCC promotes optimal cardiorespiratory transition in all newborns, especially in preterm infants who may need a longer time for this transition to happen. This allows the infant to remain hemodynamically stable and less likely to have major morbidities. Even though the individual morbidities were not different between the 60 s and 120 s DCC group, it had a favorable effect on the composite outcome of survival without major morbidities. DCC and other perinatal interventions, including ANS, antenatal magnesium for neuroprotection, and preventing hypothermia on admission, improve preterm neonatal outcomes. A Canadian neonatal network study showed the cumulative effect of ANS, magnesium for neuroprotection, DCC and normothermia on preterm outcomes: any two or more of these interventions increased survival without severe neurological injuries [27]. During the study period, we have (>95%) high antenatal steroid use, maternal magnesium use (70–77%), and no hypothermia. We observed an improvement in survival without major morbidities, increasing the duration of DCC with other optimal perinatal interventions.

Current NRP recommendations for preterm infants include 30–60 s DCC. The majority of very preterm infants start breathing 60 s after birth [28], and a longer duration of DCC is more beneficial to reduce mortality [7]. Our study adds to the growing evidence of the benefits of longer DCC. Based on our experience, 75% of preterm infants can receive a longer duration of DCC with a pragmatic approach to provide ≥ 120 s of DCC in those infants who are breathing by 60 s. The most recent VentFirst trial has shown the benefit of ≥120 s of DCC while providing positive pressure support in infants who are not breathing by 30–60 s [22]. Having a system to provide positive pressure during DCC will facilitate the implementation of longer DCC. The new evidence and further studies to compare different durations of DCC will help inform guideline and policy development regarding DR resuscitation in preterm infants.

### 4.6. Strengths and Limitations

DCC was implemented in a clinical setting with multiple obstetric and pediatric providers and trainees at a county hospital. We have clearly defined criteria for when to discontinue DCC with a collaborative approach between obstetricians and pediatricians in the delivery room. This makes it adaptable to clinical settings and generalizable to the patient population with optimal perinatal interventions, including ANS use. Study limitations include the study being performed at a single center with a small sample size and being observational, which may be subject to selection bias. Another limitation is that there may have been unintended or unmeasured practice changes over the 5 year study period, which might influence the outcomes. Study findings need to be further validated in a larger patient population across multiple centers.

## 5. Conclusions

This study shows that a longer duration of DCC for at least 120 s is feasible in the majority of the very preterm births who are breathing by 60 s of life, and this long duration is associated with an increase in survival without major morbidities. A standardized protocol and collaborative approach between obstetric and pediatric teams is essential to safely implement longer DCC. Our findings should be confirmed in larger multicenter RCTs comparing at least 120 s DCC to 60 s DCC. Future studies should evaluate the different durations of DCC with and without respiratory support to identify the optimal cord management strategy to improve preterm outcomes.

## Figures and Tables

**Table 1:** Demographics.

	Intent-to-Treat			Per-Protocol		
Study Groups	Period 1 DCC ≥ 60 s	Period 2 DCC ≥ 120 s	*p* Value	Period 1 DCC ≥ 60 s	Period 2 DCC ≥ 120 s	*p* Value
*n*	139	155		106	114	
Gestational age, weeks, median (range)	31.0 (23.2 to 32.6)	30.6 (23.5 to 32.6)	0.9	31.0 (23.2 to 32.6)	31.3 (23.5 to 32.6)	0.6
<29 weeks GA, %	27	30		25	24	
Birth weight, g, median (range)	1410 (400 to 3012)	1460 (590 to 2560)	0.6	1465 (400 to 3012)	1520 (660 to 2560)	0.6
Cesarean Delivery, %	63	65	0.7	57	55	0.8
Antenatal steroids, %	95	94	0.8	97	97	0.9
Maternal magnesium, %	76	70	0.4	75	75	1.0

**Table 2:** Deferred Cord Clamping Implementation.

	Intent-to-Treat		
Study Groups	Period 1 DCC ≥ 60 s	Period 2 DCC ≥ 120 s	*p* Value
*n*	139	155	
DCC completed, %	76	74	0.6
DCC duration, seconds, median (range)	60 (0 to 145)	129 (0 to 240)	**<0.001**
0 to <60 s, %	24	14	0.04
60 to 119 s, %	72	12	**<0.001**
120 to 179 s, %	4	34	**<0.001**
≥180 s, %		40	n/a
Cord milking, %		21	n/a
Breathing before cord clamping, %		93	n/a
Reasons for not completing DCC			
Neonatal reasons, *n* (%)	13 (9%)	21 (14%)	0.3
Maternal reasons, *n* (%)	12 (9%)	10 (6%)	0.5
Other reason recorded, *n* (%)	2 (1%)	0 (0%)	0.1
No reason recorded, *n* (%)	6 (4%)	10 (6%)	0.4

**Table 3:** (**A**) Delivery Room Measures, NICU Measures and Interventions. (**B**) NICU Morbidity and Mortality.

	Intent-to-Treat				Per-Protocol			
	Period 1 DCC ≥ 60 s	Period 2 DCC ≥ 120 s	Difference [95% CI]	*p* Value	Period 1 DCC ≥ 60 s	Period 2 DCC ≥ 120 s	Difference [95% CI]	*p* Value
(**A**)								
*n*	139	155			106	114		
**Delivery Room Measures and Interventions**								
1-min APGAR < 4, %	17	7	−10 [−18 to −2]	**0.02**	8	2	−8 [−16 to 0]	**0.04**
5-min APGAR < 8, %	33	32	−1 [−12 to 10]	0.9	28	15	−5 [−16 to 7]	0.5
Hypothermia, ^A^ %	0	0			0	0		
Chest compressions, ^A^ %	1	0		0.2	0	0		1.0
Delivery room intubation, %	6	3	−3 [−8 to 2]	0.2	5	1	−5 [−11 to 2]	0.1
**NICU Measures and** **Interventions**								
Any intubation, %	18	16	−2 [−11 to 7]	0.7	16	10	−6 [−15 to 3]	0.2
Hematocrit values 0–2 h, mean (SD)	49.4 (7.2)	50.8 (7.4)	1.4 [−0.4 to 3.3]	0.1	50.1 (7.3)	50.8 (7.4)	0.6 [−1.4 to 2.7]	0.6
Hematocrit values 12–24 h, mean (SD)	50.7 (7.9)	52.6 (8.5)	1.9 [−1.2 to 4.9]	0.2	51.3 (8.1)	53.2 (8.4)	1.9 [−1.6 to 5.4]	0.3
Erythropoietin, %	11	45	33 [24 to 43]	**<0.001**	9	43	33 [22 to 44]	**<0.001**
Early RBC Transfusion, %	5	3	−2 [−7 to 3]	0.5	5	1	−5 [−11 to 2]	0.1
Any RBC Transfusion, %	19	12	−7 [−16 to 2]	0.1	18	9	−9 [−19 to −0]	0.05
(**B**)								
**NICU Mortality and** **Morbidities**								
Severe IVH, %	4	4	0 [−4 to 5]	0.9	4	3	−1 [−6 to 4]	0.6
LOS, %	5	4	0 [−6 to 4]	0.7	5	3	−2 [−7 to 3]	0.4
CLD, %	17	10	−7 [−15 to 1]	0.08	16	8	−8 [−17 to 1]	0.07
Severe ROP, %	4	3	0 [−5 to 4]	0.9	3	4	1 [−4 to 5]	0.8
NEC, %	3	1	−2 [−5 to 2]	0.4	3	2	−1 [−5 to 3]	0.6
Death, %	3	3	0 [−4 to 3]	0.9	1	1	0 [−3 to 2]	1.0
Survival without Major Morbidities, %	76	83	7 [−2 to 16]	0.1	76	88	11 [1 to 21]	0.03
**Subgroup Analysis ^B^**								
<29 weeks GA	46	53		0.5	44	59		0.3
≥29 weeks GA	87	96		0.02	87	97		0.04

A—Fischer exact test was used instead of generalized estimating equations due to the lack of events within groups. B—Subgroup analysis of survival without major morbidities. IVH—intraventricular hemorrhage, LOS—late-onset sepsis, CLD—chronic lung disease, ROP—retinopathy of prematurity, NEC—necrotizing enterocolitis, GA—gestational age.

**Table 4:** Survival without Major Morbidities (Risk-Adjusted Analysis).

	Period 1 DCC ≥ 60 s	Period 2 DCC ≥ 120 s
*n*	106	114
Survival without major morbidities, %	76	88
**Survival without major morbidities outcome models**	**Odds Ratio [95% CI]**	***p* value**
Model#1:		
DCC duration group	2.2 [1.1 to 4.5]	**0.03**
Model#2:		
DCC duration group	8.6 [1.6 to 45.7]	**0.01**
Gestational age	1.7 [1.4 to 2.0]	**<0.0001**
Erythropoietin	0.2 [0.0 to 1.1]	0.07

## Data Availability

The original data presented in the study are openly available in https://doi.org/10.5061/dryad.vt4b8gv2r.
